# The DEAD-Box RNA Helicase DDX3 Interacts with NF-κB Subunit p65 and Suppresses p65-Mediated Transcription

**DOI:** 10.1371/journal.pone.0164471

**Published:** 2016-10-13

**Authors:** Nian Xiang, Miao He, Musarat Ishaq, Yu Gao, Feifei Song, Liang Guo, Li Ma, Guihong Sun, Dan Liu, Deyin Guo, Yu Chen

**Affiliations:** 1 State Key Laboratory of Virology, College of Life Sciences, Wuhan University, Wuhan, P. R. China; 2 School of Basic Medical Sciences, Wuhan University, Wuhan, P. R. China; University of Leeds Faculty of Medicine and Health, UNITED KINGDOM

## Abstract

RNA helicase family members exhibit diverse cellular functions, including in transcription, pre-mRNA processing, RNA decay, ribosome biogenesis, RNA export and translation. The RNA helicase DEAD-box family member DDX3 has been characterized as a tumour-associated factor and a transcriptional co-activator/regulator. Here, we demonstrate that DDX3 interacts with the nuclear factor (NF)-κB subunit p65 and suppresses NF-κB (p65/p50)-mediated transcriptional activity. The downregulation of DDX3 by RNA interference induces the upregulation of NF-κB (p65/p50)-mediated transcription. The regulation of NF-κB (p65/p50)-mediated transcriptional activity was further confirmed by the expression levels of its downstream cytokines, such as IL-6 and IL-8. Moreover, the binding of the ATP-dependent RNA helicase domain of DDX3 to the N-terminal Rel homology domain (RHD) of p65 is involved in the inhibition of NF-κB-regulated gene transcription. In summary, the results suggest that DDX3 functions to suppress the transcriptional activity of the NF-κB subunit p65.

## Introduction

DEAD-box RNA helicases are involved in many aspects of RNA metabolism, including transcription, pre-mRNA processing, RNA decay, ribosome biogenesis, RNA export and translation [1]. Deactivation of the same subfamilies of DEAD-box RNA helicases, such as DDX3, consistently results in disease [2]. The DEAD-box protein family contains conserved motifs: ATPase motif I: responsible for ATP hydrolysis; motif II (Asp-Glu-Ala-Asp, D-E-A-D): binds the nucleotide triphosphate-Mg^2+^ complex; motif III: an ATP-dependent helicase domain that unwinds RNA duplexes; and motifs Ia, Ib, IV and VI: RNA binding [1].

DDX3 is a DEAD-box RNA helicase with eight conserved helicase domains that is expressed in multiple tissues, ranging from blood to brain cells [3]. DDX3 functions in multiple biological processes, including RNA metabolism, the RNA interference (RNAi) pathway, viral replication, cell cycle, the innate immune response, the regulation of gene expression, and tumourigenesis as both a tumour suppressor and a promoter [3–7]. Surprisingly, DDX3 is a component of the innate immune response against viral infection. Moreover, several RNA viruses, such as HIV-1 and hepatitis C virus (HCV), use DDX3 to accomplish viral replication by exporting viral RNA and manipulating transcriptional and translational regulation [8–10]. More importantly, DDX3 directly interacts with the p21^waf1/cip1^ (a cyclin-dependent kinase inhibitor) promoter through its four SP1 sites (located within the -123 to -63 region) and utilizes ATPase-dependent activity to inhibit the colony formation ability of various tumour cells [11–14]. Moreover, DDX3 increases p53 accumulation and positively regulates DNA damage-induced apoptosis [15]. However, the loss of DDX3 by p53 inactivation promotes malignancy [16]. Many of these functions are associated with transcriptional co-activation or regulation by DDX3. Interestingly, other RNA helicases, such as RNA helicase A, the DEAD-box protein DP103 and p6, also act as transcriptional co-activators/regulators to perform multiple physiological functions [17–22]. Therefore, it is critical to gain insight into the transcriptional regulatory role of DDX3, an important DEAD-box RNA helicase.

Nuclear factor kappa B (NF-κB) regulates genes associated with tumourigenesis/carcinogenesis, tumour suppression, inflammation, proliferation, apoptosis, immune regulation and viral manipulation [23–30]. NF-κB suppresses apoptosis and promotes cancer development by regulating the expression of anti-apoptotic genes, such as Bcl-XL, IAP (inhibitors of apoptosis), and cFLIP [31]. Studies of NF-κB in cancer have focused on its induction of apoptosis resistance and its role in carcinogenesis [32]. Five members of the NF-κB family have been identified (c-Rel, p65 [RelA] [6], NFκB1 [p50/p105], RelB and NFκB2 [p52/p100]), and these proteins form hetero- and homodimers with distinct specificity for transcriptional activation [33]. In unstimulated cells, NF-κB/Rel proteins are bound and inhibited by IκB proteins. In the classical (or canonical) pathway, inducers, such as cytokines and tumour necrosis factor α (TNFα), activate an IKK complex (IKKβ, IKKα, and NEMO) that induces the phosphorylation and degradation of NF-κB inhibitor (IκB) proteins. IκB subsequently releases activated NF-κB (p65/p50), which translocates into the nucleus and induces target gene expression [34]. The activator/co-activator role of NF-κB in transcriptional activation is well studied; however, little work has been performed to determine the function of repressors in regulating NF-κB transactivation. In this study, to gain more insight into the negative regulation of NF-κB activity, we evaluated the effect of the tumourigenesis-associated factor DDX3 on transcriptional factors involved in the NF-κB signalling pathway. We demonstrated that DDX3 binds to p65 to exert a strong inhibitory effect on NF-κB (p65/p50)-mediated transcriptional activity.

## Materials and Methods

### Plasmids and reagents

The pM-DDX3 and pVP16-p65 plasmids were generated by cloning human DDX3 cDNA [10] fused to the Gal4 DNA binding domain (DBD) in the pM vector and p65 cDNA [35] fused to the transcriptional activation domain in pVP16 (Clontech, USA). A plasmid containing pFlag-DDX3 under control of the cytomegalovirus (CMV) promoter was obtained by cloning the respective cDNA into the pCMV2A vector (Stratagene, USA). The pHA-DDX3, pHA-DDX3 (1–310), pHA-DDX3 (310–662) and pHA-DDX3-K230E plasmids were described previously [10]. The pHA-p65 (FL), pHA-p65 (1–322) and pHA-p65 (299–551) plasmids were obtained from Dr Jonathan D. Licht [35]. The expression plasmids for IKKβ, c-Rel and NIK, as well as the luciferase reporter plasmids p5xNF-κB-luc, pRL-TK and pELAM-luc, were kindly provided by Dr Hong-Bing Shu [36, 37]. Mouse monoclonal antibodies against Flag (M2) and HA were purchased from Sigma. DDX3 N-terminal polyclonal antibodies and mouse monoclonal β-actin antibodies were obtained from Abcam. A mouse monoclonal antibody against p65 was purchased from Santa Cruz Biotechnology. Mouse polyclonal antibodies against LMNB1 and rabbit polyclonal antibodies against β-tubulin were purchased from Proteintech. The siRNA products were purchased from RiboBio.

To analyse whether the enzymatic domain (ATPase/helicase) of DDX3 is required, we created point mutations in the conserved “PTRELAVQ” motif by independently mutating threonine (T) and glutamic acid (E) at positions 275 and 277 to arginine (R) and valine (V) (pHA-DDX3-T275R/E277V). The polymerase chain reaction (PCR)-generated point mutant forms of DDX3 gene fragments were cloned into an HA-tag vector [35].

### Luciferase reporter activity assay

HEK293T (293T) cells were grown in Dulbecco’s modified Eagle medium supplemented with 10% foetal bovine serum (FBS) (HyClone, USA). Luciferase activity was measured 24 hours after transfection using a Dual-Luciferase Reporter Assay System (Promega, Madison, WI) as described in the manual. Relative luciferase activity was obtained by comparing various protein expression constructs with empty vector.

### Immunoprecipitation

To examine protein-protein interactions, 293T cells were cultured in 10-cm^2^ dishes and transfected with 4 to 8 μg of the indicated combinations of expression plasmids. Cell lysates were incubated with an anti-Flag or anti-HA antibody overnight at 4°C. Immunocomplexes were captured by incubation with protein A/G-agarose beads for 2 hours at 4°C. The immunoprecipitated proteins were resolved by 10% sodium dodecyl sulphate-polyacrylamide gel electrophoresis (SDS-PAGE) and analysed by Western blotting with anti-HA or anti-Flag antibodies. For endogenous immunoprecipitations, non-transfected cell lysates were incubated with polyclonal anti-DDX3 antibodies or IgG (control). Western blotting was performed using anti-p65 and anti-DDX3 antibodies to detect endogenous p65 and DDX3 proteins.

### Indirect immunofluorescence analysis

To determine the co-localization of p65 and DDX3, 293T cells were immunostained with rabbit polyclonal anti-DDX3 and mouse monoclonal anti-p65 primary antibodies. Immunofluorescence analysis of the intracellular localization of red and green fluorescence was performed using confocal microscopy as described previously [38].

### RNAi knockdown of DDX3

To knock down endogenous DDX3, an effective target site was used for RNA interference (RNAi). After 24 hours of short hairpin RNA (shRNA) or small interfering RNA (siRNA) transfection, cells were transfected with a luciferase reporter plasmid. The transfected cells were harvested after 24 hours and lysed in SDS sample buffer for Western blotting or in passive lysis buffer (Promega, USA) for luciferase activity assays.

### Protein purification and GST pull-down

The corresponding plasmids were transformed into *E*. *coli* BL21 (DE3) and expressed. GST and recombinant GST-tagged DDX3 (GST-DDX3) were purified using glutathione resin beads (GenScript). Recombinant His-tagged p65 (His-p65) was purified using Ni^2+^-NTA resin (Novagen) according to the instruction manual. The eluted proteins were quantitated, and 50 ng each of GST-DDX3 and GST was immobilized on glutathione resin beads in the rotating incubator at 4°C. After incubation for 2 hours, the beads were collected and washed 3 times with N-lysis buffer (0.15 mM NaCl, 1% NP-40, 50 mM Tris-HCl, pH 8.0, and 5 mM EDTA). Then, an equal amount of input His-p65 protein (50 ng) was added to the beads, and the mixture was incubated for 4 hours. After removing the supernatant, the beads were collected and washed 6 times with N-lysis buffer. Each sample of beads bound to target protein was analysed by SDS-PAGE and Western blotting.

### Cellular fractionation

The cytoplasmic and nuclear extracts were obtained using a nuclear-cytoplasmic extraction kit (Applygen Technologies) according to the manufacturer’s instructions.

### Quantitative RT-PCR

Total RNA was extracted from HepG2 cells with Trizol reagent (Invitrogen) and was reverse transcribed with a Reverse Transcription System (Promega); then, total cDNA was amplified in an ABI 7500 Detection System (Applied Biosystems) using SYBR Green PCR Master Mix (Roche). The primers were as follows: IL-6 sense 5'-AGACAGCCACTCACCTCTTCAG-3' and antisense 5'-TTCTGCCAGTGCCTCTTTGCTG-3'; IL-8 sense '5'-GAGAGTGATTGAGAGTGGACCAC-3 and antisense 5'- CACAACCCTCTGCACCCAGTTT-3'; and GAPDH sense 5'-CCACCCATGGCAAATTCCATGGCA-3' and antisense 5'-TCTAGACGGCAGGTCAGGTCCACC-3'. The expression of the GAPDH control gene was used to normalize target gene expression in each sample. The relative expression levels of the target genes were calculated using the 2^−ΔΔCt^ method.

### Measurement of IL-6 and IL-8 in cultured cell supernatants

Thirty-six hours post transfection, HepG2 cells were stimulated with TNF-α. IL-6 and IL-8 levels in the HepG2 culture supernatants were measured using enzyme-linked immunosorbent assay (ELISA) kits (Abcam, UK) according to the manufacturer’s instructions.

## Results

### Suppression of NF-κB-mediated transcription by DDX3

**Fig 1:**
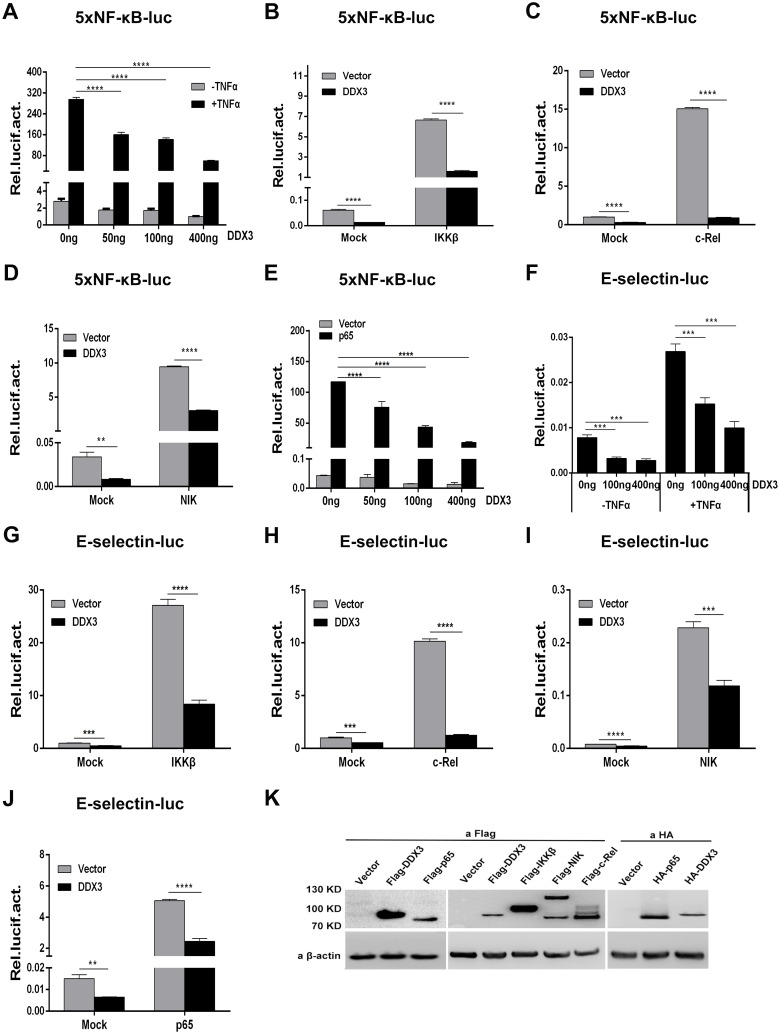
Suppression of TNFα-, IKKβ-, c-Rel, NIK- and p65-induced NF-κB transcriptional activity by DDX3. DDX3 inhibits TNFα-, IKKβ-, c-Rel-, NIK- and p65-induced NF*-*κB activation. 293T cells were co-transfected with reporter plasmid (50 ng), *Renilla* luciferase control plasmid pRL-TK (20 ng) and pHA-DDX3 (400 ng or the indicated amount) or the empty vector pcDNA3.0 (400 ng) as a negative control. Twenty-four hours post transfection, firefly and *Renilla* luciferase activity was determined. (A to E) The 5xNF-κB luciferase reporter (5xNF-κB-luc) was activated by TNFα (20 ng/ml) or 100 ng of IKKβ, c-Rel, NIK or p65. (F to J) The E-selectin luciferase reporter (E-selectin-luc) was activated by TNFα (20 ng/ml) or 100 ng of IKKβ, c-Rel, NIK or p65. The mock (non-induced) control was transfected with 100 ng of empty vector corresponding to each inducing factor. The data are plotted as the mean ± standard deviation (*n* = 3, mean ± SD). ** p < 0.01, *** p < 0.001 and **** p < 0.0001 (unpaired Student’s t-test). (K) The expression of all the indicated genes was confirmed by Western blotting using the indicated antibodies.

To investigate the role of DDX3 in NF-κB transcriptional activity, we used an NF-κB-dependent luciferase reporter system in 293T cells. In the NF-κB luciferase reporter assay, TNFα, IKKβ, c-Rel, NIK and p65 each induced the expression of a reporter gene (Fig 1). TNFα-induced 5xNF-κB-luc reporter activity decreased in a dose-dependent manner when DDX3 was co-expressed (Fig 1A). In addition, 5xNF-κB-luc activity induced by IKKβ, c-Rel or NIK was significantly inhibited by the expression of DDX3 (Fig 1B to 1D). Interestingly, p65-induced 5xNF-κB-luc reporter activity was also inhibited in a dose-dependent manner by DDX3 co-expression (Fig 1E). Moreover, DDX3 expression significantly inhibited the transcriptional activity of the physiological E-selectin-luciferase (pELAM-luc) reporter system harbouring NF-κB binding sites in a dose-dependent manner with or without TNFα induction (Fig 1F). Unsurprisingly, pELAM-luc reporter activity induced by IKKβ, c-Rel, NIK or p65 was similarly inhibited by DDX3 expression (Fig 1G to 1J). The expression of all the above-mentioned factors was confirmed by Western blotting (Fig 1K). These results indicated that DDX3 specifically suppresses NF-κB-mediated transcription.

### Promotion of NF-κB-mediated transcriptional activity by knockdown of endogenous DDX3

**Fig 2:**
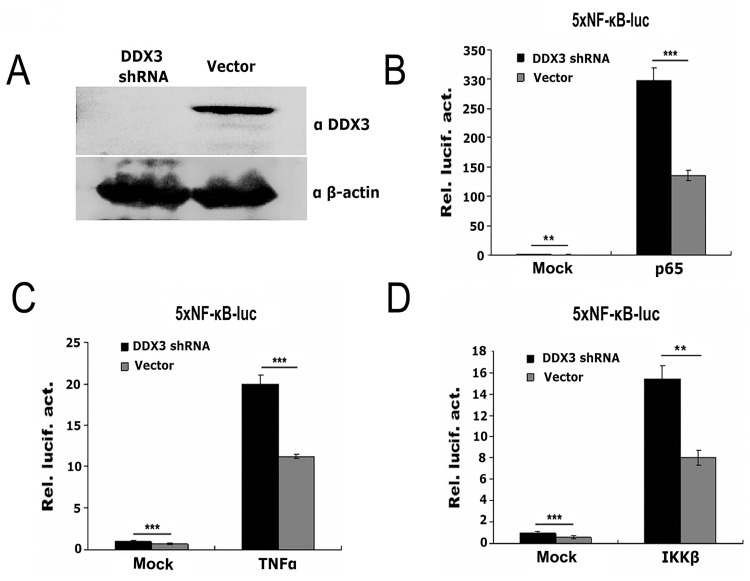
Silencing of endogenous DDX3 by shRNA results in the upregulation of NF-κB-mediated transcriptional activity. (A) Inhibition of endogenous DDX3 protein expression by a DDX3-specific shRNA plasmid in 293T cells. An empty vector was used as a negative control. DDX3 was detected by anti-DDX3 antiserum (upper panel). β-Actin was used as an internal control, indicating that equal amounts of total protein were loaded (lower panel). (B to D) Knockdown of endogenous DDX3 upregulates NF-κB transcriptional activity induced by p65, TNFα or IKKβ (*n* = 3, mean ± SD). ** p < 0.01 and *** p < 0.001 (unpaired Student’s t-test). The mock (non-induced) control was transfected with 100 ng of empty vector corresponding to each inducing factor.

**Fig 3:**
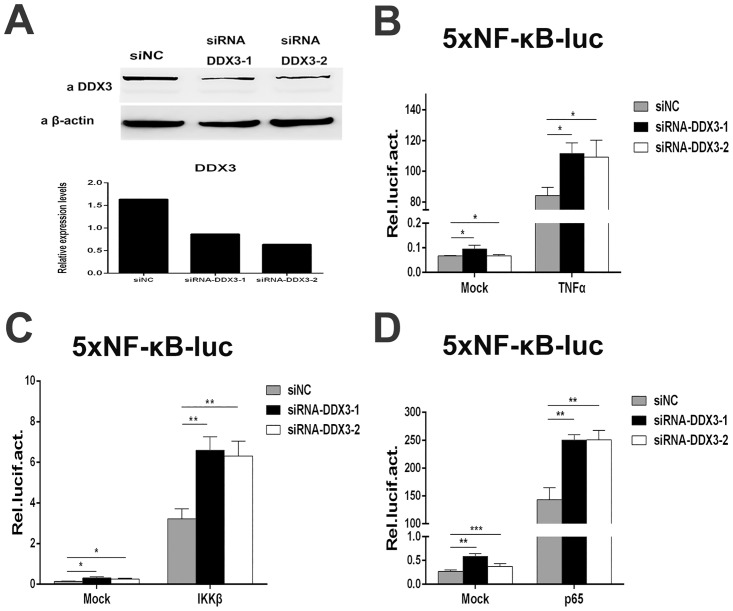
Silencing of endogenous DDX3 by siRNA increases NF-kB-mediated transcriptional activity. (A) Inhibition of endogenous DDX3 protein expression by two different DDX3-specific siRNAs in 293T cells. An unrelated siRNA (siNC) was used as a negative control. DDX3 was detected using an anti-DDX3 antibody, and β-actin served as an internal control (upper panel). The relative expression levels of endogenous DDX3 compared to β-actin are quantified in the lower panel. (B to D) Knockdown of endogenous DDX3 upregulates NF-κB-mediated transcriptional activation induced by TNFα, IKKβ or p65 (n = 3, mean ± SD). * p < 0.05, ** p < 0.01 and *** p < 0.001 (unpaired Student’s t-test). The mock (non-induced) control was transfected with 100 ng of empty vector corresponding to each inducing factor.

To investigate the physiological effect of endogenous DDX3 on the NF-κB pathway, DDX3 expression was knocked down by DDX3-specific shRNA (Fig 2) and siRNA (Fig 3). As shown in Fig 2A, endogenous DDX3 protein levels were significantly downregulated by transfection with DDX3-specific shRNA, whereas β-actin levels were not affected (Fig 2A). The NF-κB transcriptional activity induced by p65, TNFα or IKKβ was significantly increased in cells treated with DDX3 shRNA compared with control vector (Fig 2B to 2D). To exclude off-target effects of DDX3 shRNA, two individual DDX3-specific siRNAs (siRNA DDX3-1 and DDX3-2) were designed and subjected to the functional tests (Fig 3). Endogenous DDX3 expression was significantly knocked down by the DDX3-specific siRNAs (Fig 3A); DDX3 expression levels were quantified and normalized to those of β-actin (Fig 3A). The NF-κB transcriptional activity induced by TNFα, IKKβ or p65 was significantly increased after treatment with DDX3-specific siRNAs (Fig 3B to 3D). This result demonstrated that knockdown of endogenous DDX3 significantly increased NF-κB-mediated transcriptional activity.

### Identification of p65 as a DDX3-interacting protein

**Fig 4:**
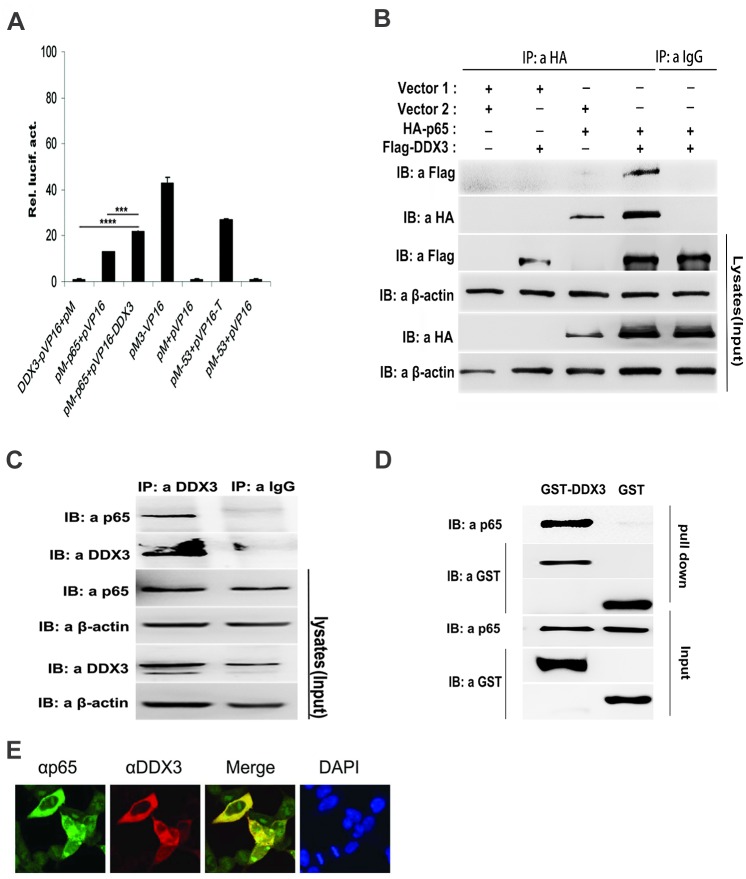
Interaction of DDX3 with the NF-κB p65 subunit. (A) DDX3 specifically interacts with p65 in a mammalian two-hybrid system. Here, 293T cells were co-transfected with Gal4-luc reporter plasmid (100 ng) and an equal amount of pVP-DDX3 and pM-p65 (350 ng) or various control plasmids. Luciferase activity was determined 48 hours post transfection (*n* = 3, mean ± SD). *** p < 0.001 and **** p < 0.0001 (unpaired Student’s t-test). (B) Immunoprecipitation of Flag-tagged DDX3 and HA-tagged p65. 293T cells were co-transfected with Flag-DDX3, HA-p65 or empty vector. HA-tagged proteins were immunoprecipitated with an anti-HA antibody and immunoblotted with the Flag antibody to detect the presence of Flag-tagged DDX3 (co-IP) (upper layer). The HA antibody was used to detect the IP fraction (middle layers). Anti-HA or anti-Flag antibody was used to detect HA-p65 or Flag-DDX3, respectively, in cell lysates (input, lower layers). (C) Immunoprecipitation of endogenous DDX3 and p65. Lysates from non-transfected 293T cells were immunoprecipitated with either DDX3 polyclonal antibody or IgG. Western blotting with mouse monoclonal p65 antibody detected the presence of endogenous p65 (co-IP) (upper layer). The DDX3 polyclonal antibody was used to detect the IP fraction (middle layers). The presence of similar amounts of DDX3 and p65 in the lysates is shown in the lower panels. (D) GST pull-down assay. Purified GST-DDX3 or GST protein was incubated with His-p65 and pulled down with glutathione resin beads. Each sample was detected by Western blotting with the indicated antibodies. GST protein was used as a negative control. (E) Indirect immunofluorescence was performed on non-transfected 293T cells to determine the endogenous co-localization of p65 and DDX3 using specific anti-p65 and anti-DDX3 antibodies. Merged images (yellow) of DDX3, p65 and DAPI nuclear staining (blue) are presented.

Over-expression or knockdown of DDX3 significantly suppressed or enhanced NF-κB transcriptional activity induced by TNFα, IKKβ and p65. Moreover, TNFα and IKKβ are upstream of p65, which is located at the end of the NF-κB signalling pathway. The results indicated that DDX3 might disturb the NF-κB signalling pathway downstream of p65. NF-κB subunit p65 (RelA), a transcription factor, is involved in the activation of multiple genes. By screening, we found that DDX3 interacts with p65. As shown in Fig 4A, the co-transfection of 293T cells with DDX3 fused to the VP16 activation domain (pVP16-DDX3) and p65 fused to the Gal4 DBD (pM-p65) significantly activated luciferase reporter gene expression, similar to the positive controls (pM3-VP16 or the combination of pM53 and pVP16-T). However, DDX3 or p65 alone, as well as various negative controls, could not activate luciferase reporter gene expression. To confirm this interaction, 293T cells were transfected with Flag-tagged DDX3 and HA-tagged p65 expression plasmids. HA-p65 co-immunoprecipitated with Flag-DDX3. In contrast, a significant HA-p65 immunocomplex was not observed after transfection with Flag-DDX3 or HA-p65 and empty vector as a negative control (Fig 4B). Moreover, endogenous p65 was immunoprecipitated using a rabbit polyclonal DDX3 antibody and detected by a mouse monoclonal anti-p65 antibody, suggesting that endogenous p65 could associate with DDX3 (Fig 4C). To further investigate if this interaction is direct or indirect, recombinant GST-tagged DDX3 (GST-DDX3) and His-tagged p65 (His-p65) were expressed in *E*. *coli* and purified. In the GST pull-down assay, purified His-p65 co-immunoprecipitated with GST-DDX3, indicating that DDX3 directly interacts with p65 (Fig 4D). DDX3 is primarily localized in the cytoplasm and acts as a shuttling protein [10]. The subcellular co-localization of endogenous p65 and DDX3 *in vivo* further confirmed that DDX3 could associate with p65 in the cytoplasm (Fig 4E). Taken together, these results indicate that the RNA helicase DDX3 is a cellular interaction partner of the NF-κB subunit p65 and negatively regulates the transcriptional activity of the NF-κB signalling pathway.

### Mapping the interaction domain of p65 and the suppression domain of DDX3

**Fig 5:**
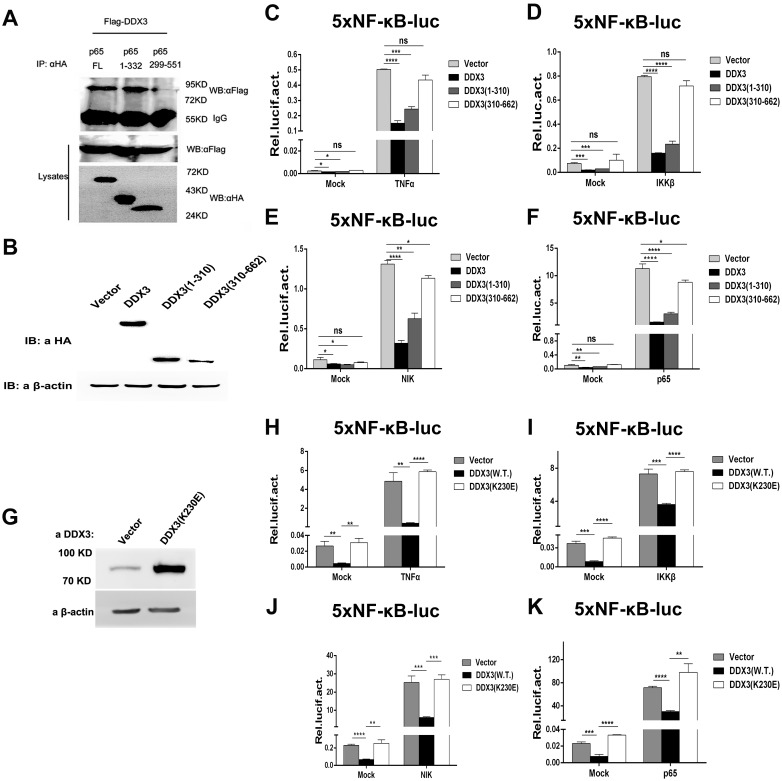
The Rel homology domain of p65 specifically binds to DDX3, and the ATP-dependent RNA helicase activity of DDX3 is important for its inhibitory effect on NF-κB target gene expression. (A) Co-immunoprecipitation of HA-tagged p65 wt and deletion mutants. Lysates of 293T cells transfected with Flag-DDX3 (4 μg) and HA-p65 wt or deletion mutants (1–332 or 299–551) (4 μg) were used for immunoprecipitation analysis. Anti-HA antibodies were used to capture the immunocomplex. DDX3 in the immunoprecipitates was detected by immunoblotting with mouse monoclonal anti-Flag antibodies. Anti-Flag and anti-HA antibodies were used to detect Flag-DDX3 and HA-p65 wt and deletion mutants (1–332 or 299–551), respectively, in whole cell lysates (middle and lower layers). (B) Truncated DDX3 (1–310) and DDX3 (310–662) expression levels were detected by Western blotting. (C to F) Effect of truncated DDX3 on NF-κB-mediated gene expression. The 5xNF-κB-luc reporter gene was activated by TNFα (20 ng/ml) or 100 ng of IKKβ, NIK or p65. The cells were co-transfected with 400 ng of DDX3, DDX3 (1–310), DDX3 (310–662) or empty vector (pcDNA3.0, negative control). Firefly and *Renilla* luciferase activity was measured (*n* = 3, mean ± SD) at 24 hours post transfection. (G) The expression level of DDX3-K230E was detected by Western blotting. (H to K) Effect of the ATP-dependent RNA helicase mutant of DDX3 on NF-κB-mediated gene expression. The 5xNF-κB-luc reporter gene was activated by TNFα (20 ng/ml) or 100 ng of IKKβ, NIK or p65. The cells were co-transfected with 400 ng of wild-type or mutant DDX3 expression plasmid (HA-DDX3-K230E) or empty vector (pcDNA3.0, negative control). Firefly and *Renilla* luciferase activity was measured (*n* = 3, mean ± SD) at 24 hours post transfection. ** p < 0.01, *** p < 0.001 and **** p < 0.0001 (unpaired Student’s t-test). The mock (non-induced) control was transfected with 100 ng of empty vector corresponding to each inducing factor.

NF-κB subunit p65 contains two specific domains: an N-terminal domain, also called the Rel homology domain (RHD), that is responsible for dimerization and DNA binding, and a C-terminal transcriptional activation domain [39]. To identify the interaction domain of p65, HA-tagged wild type (wt) p65 (pHA-p65) [35] and two truncation mutants, pHA-p65 (amino acids 1–332, RHD) and pHA-p65 (amino acids 299–551), were each co-transfected with Flag-tagged DDX3 (pFlag-DDX3) into 293T cells. As shown in Fig 5A, both wt p65 and the DNA-binding domain (RHD) of p65 pulled down Flag-tagged DDX3 via a mouse monoclonal anti-HA antibody and were significantly detected by the Flag monoclonal antibody. However, the C terminus of p65 (amino acids 299–551) failed to co-immunoprecipitate with DDX3 (Fig 5A). These results indicate that the inhibitory effect of DDX3 is associated with the RHD of p65.

**Fig 6:**
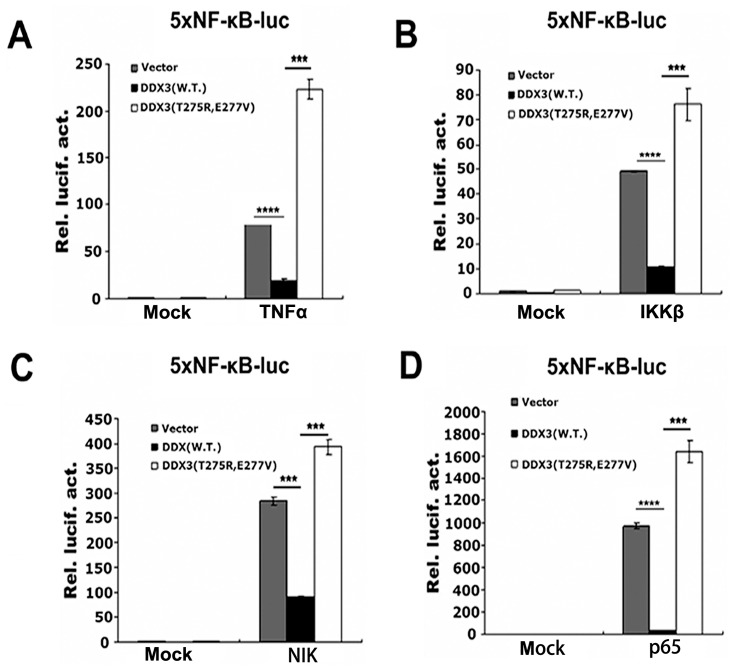
Double mutation of DDX3 (HA-DDX3-T275R/E277V) disables the inhibitory effect on NF-κB target gene expression. (A to D) The 5xNF-κB-luc reporter gene was activated by TNFα (20 ng/ml) or 100 ng of IKKβ, NIK or p65. 293T cells were co-transfected with 400 ng of wild-type or mutant DDX3 expression plasmid (HA-DDX3-T275R/E277V) or empty vector (pcDNA3.0, negative control). Firefly and *Renilla* luciferase activity was measured (*n* = 3, mean ± SD) at 24 hours post transfection. *** p < 0.001 and **** p < 0.0001 (unpaired Student’s t-test). The mock (non-induced) control was transfected with 100 ng of empty vector corresponding to each inducing factor.

To further define the region of DDX3 involved in the NF-κB signalling pathway, we truncated DDX3 into the N-terminal ATP-dependent RNA helicase domain (amino acids 1–310) and the C-terminal domain (amino acids 310–662) and fused the truncated proteins to an N-terminal HA tag (Fig 5B). In the NF-κB reporter assay induced by TNFα, IKKβ, NIK or p65 in 293T cells, the ATP-dependent RNA helicase domain of DDX3 (1–310) markedly repressed NF-κB-mediated transcriptional activity similar to wt DDX3. However, DDX3 (310–662) did not show significant inhibitory activity compared to DDX3 (1–310) and wt DDX3 (Fig 5C to 5F). We further generated a single mutation within the conserved ATP-dependent RNA helicase motif of DDX3 (HA-DDX3-K230E), which was reported as an ATP-dependent RNA helicase dominant-negative mutant that can inhibit the RNAi pathway [6, 10]. The expression of HA-DDX3-K230E was confirmed by Western blotting (Fig 5G). In the NF-κB reporter assay induced by TNFα, IKKβ, NIK or p65 in 293T cells, DDX3 harbouring a single point mutation (HA-DDX3-K230E) lost its inhibitory effect on NF-κB-mediated transcriptional activity compared with wt DDX3 (Fig 5H to 5K). Furthermore, we designed a double mutation (HA-DDX3-T275R/E277V) within the conserved ATP-dependent RNA helicase motif of DDX3 based on bioinformatics; the targeted residues are essential for ATP-dependent RNA helicase activity. In the NF-κB reporter assay induced by TNFα, IKKβ, NIK or p65 in 293T cells, HA-DDX3-T275R/E277V exhibited the opposite activity as wt DDX3, significantly activating reporter activity compared to DDX3 (Fig 6). Taken together, the results suggest that the ATP-dependent RNA helicase domain of DDX3 regulates NF-κB (p65/p50)-mediated target gene transcription by interacting with the N-terminal RHD of p65.

### DDX3 binds p65 in the cytoplasm and represses the expression of IL-6 and IL-8

**Fig 7:**
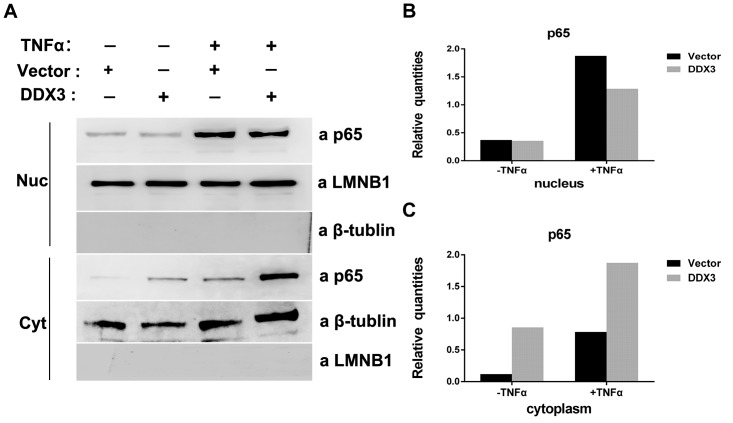
DDX3 binds p65 in the cytoplasm. (A) Immunoblotting analysis of 293T cells transfected with vector (2 μg) or a DDX3 expression plasmid (2 μg). The indicated cells were treated with TNFα (20 ng/ml) 24 hours post transfection. The nuclear and cytoplasmic extracts were obtained 30 hours post transfection. LMNB1 and β-tubulin were used as nuclear and cytoplasmic controls, respectively. (B and C) The levels of p65 in each fraction were quantified and normalized to those of LMNB1 or β-tubulin.

**Fig 8:**
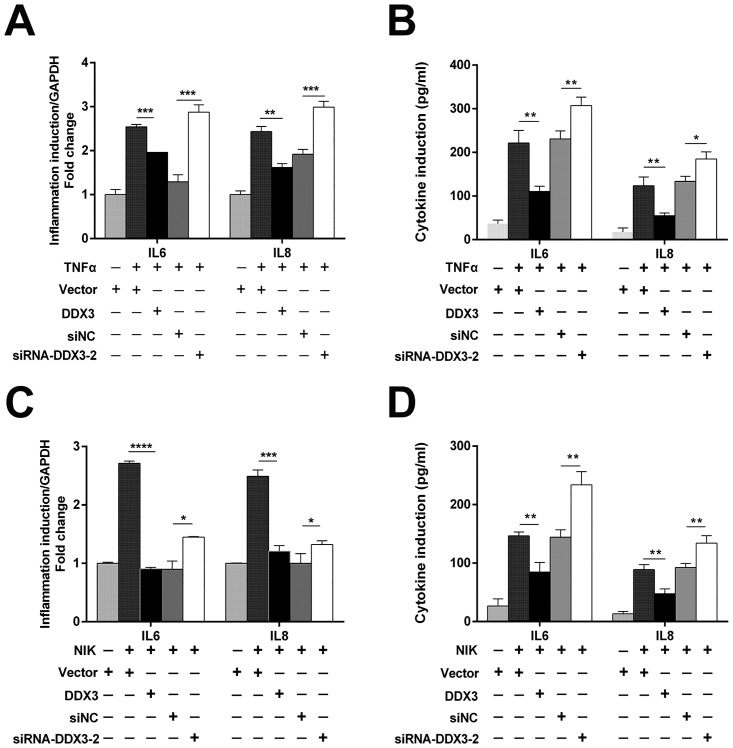
Over-expression or knockdown of DDX3 regulates the expression levels of IL-6 and IL-8. HepG2 cells were transfected with control vector (1 μg), DDX3 expression plasmid (1 μg), negative control siRNA (siNC) (50 nm) or siRNA-DDX3-2 (50 nm) as indicated. (A and B) At 24 hours post transfection, the indicated cells were stimulated with TNFα (20 ng/ml) for 6 hours. The mRNA (A) and protein (B) levels of IL-6 and IL-8 were detected by real-time PCR and ELISA, respectively. GAPDH mRNA levels served as an internal control. (C and D) HepG2 cells were stimulated by the co-transfection of NIK expression plasmid (0.2 μg). At 24 hours post transfection, the mRNA (C) and protein (D) levels of IL-6 and IL-8 were detected by real-time PCR and ELISA, respectively. *n* = 3, mean ± SD. * p < 0.05, ** p < 0.01, *** p < 0.001 and **** p < 0.0001 (unpaired Student’s t-test).

To further investigate the consequence of the interaction between DDX3 and p65, DDX3 and its vector control were transfected into 293T cells. After treatment with or without TNFα, nuclear and cytoplasmic extracts were obtained and subjected to Western blotting. After TNFα treatment, DDX3 transfection slightly reduced the amount of nuclear p65 compared with vector control (Fig 7); however, there was significant accumulation of cytoplasmic p65 (Fig 7). We further measured the mRNA and protein levels of the NF-κB downstream cytokines IL-6 and IL-8 in HepG2 cells. As shown in Fig 8A and 8B, upon TNFα treatment, the mRNA and protein levels of IL-6 and IL-8 were significantly repressed by DDX3. In contrast, knocking down DDX3 with siRNA-DDX3 markedly increased the mRNA and protein levels of both IL-6 and IL-8. The same phenomena were observed when the NF-κB signalling pathway was activated by NIK transfection (Fig 8C and 8D). Based on these results, we speculate that DDX3 directly interacts with p65 in the cytoplasm, which results in the downregulation of NF-κB (p65/p50)-mediated transcription and the subsequent reduction in IL-6 and IL-8 expression.

## Discussion

NF-κB is a key cellular transcription factor that controls the expression of more than 500 genes [28, 40, 41]. Dysfunction within the NF-κB signalling pathway leads to various diseases [24, 27, 42]. Moreover, constitutive activation of NF-κB has been observed in multiple solid tumours; this provides oncogenic signals to cancer cells, resulting in malignant behaviour [26, 28, 32]. Therefore, regulation of the NF-κB signalling pathway is important for cells. In particular, negative regulatory factors are essential to avoid over-stimulation of this complex signalling pathway [43]. Furthermore, elucidation of the mechanisms underlying the negative regulation of the NF-κB signalling pathway will provide potential strategies for the therapeutic treatment of NF-κB-associated cancer. Here, we demonstrate that DDX3 interacts with the NF-κB subunit p65 and negatively regulates NF-κB (p65/p50)-mediated transcriptional activity, which subsequently attenuates the expression of downstream genes, such as IL-6 and IL-8. However, more evidence is required to confirm if the interaction between DDX3 and p65 is direct or indirect. This study provides evidence for the involvement of DDX3 in tumour development. However, it is notable that both DDX3 and NF-κB are reported as tumourigenesis-associated factors involved in both carcinogenesis and tumour suppression. We explored the effects of DDX3 knockdown and over-expression on proliferation and invasion (S1 Fig). Surprisingly, over-expression of DDX3 promoted the proliferation/viability of HCT116 cells (colon cancer cell line) in a CCK-8 assay (S1A Fig). In contrast, siRNA-mediated knockdown of endogenous DDX3 reduced the proliferation/viability (S1B Fig) and migration of HCT116 cells in transwell assays (S1C Fig). These data imply that tumourigenesis is a complex biological process and that DDX3 might affect tumourigenesis at multiple levels. Therefore, additional studies and more evidence for the role of DDX3 in the NF-κB signalling pathway are required.

In addition to its functions in tumourigenesis [11, 13], DDX3 is involved in RNA metabolism, transcription, splicing, mRNA nuclear export, translation, RNA decay, the RNAi pathway and ribosome biogenesis [4, 6]. Furthermore, the DDX3 RNA helicase belongs to the DEAD-box family and contains ATP-dependent RNA helicase activity [10, 44]. The DEAD-box RNA helicases p68 and p72 potentially act as transcriptional co-activators for oestrogen-receptor alpha (ERα) and the tumour suppressor p53 [20]. However, these proteins also interact with histone deacetylase I (HDACI) and act as promoter-specific transcriptional repressors [45]. In this study, we demonstrated that the conserved ATP-dependent RNA helicase domain of DDX3 is involved in suppressing NF-κB (p65/p50)-mediated transcriptional activation. Both the enzymatic inactive mutant HA-DDX3-K230E and the ATP-dependent RNA helicase motif mutant HA-DDX3-T275R/E277V significantly stimulated NF-κB transcriptional activity (Figs 5 and 6). However, DDX3 also plays a role in the RNAi pathway; the mutant DDX3-K230E can inhibit this pathway [6], which is involved in widespread post-transcriptional gene regulation. Therefore, one possibility is that the mutants could not interact with p65 or could not directly inhibit NF-κB-mediated transcriptional activity. Another potential explanation is that the DDX3-K230E mutant inhibited the effects on the RNAi pathway and indirectly inhibited NF-κB-mediated transcriptional activity. Although the mechanism remains unknown, these results strongly suggest that the ATP-dependent RNA helicase activity of DDX3 is involved in the suppression of p65 transcriptional activity.

The putative role of DDX3 suggests that this multifunctional protein might connect the NF-κB signal transduction cascade with cellular functions, such as nuclear transport, RNAi, RNA metabolism and cell growth. Given that DDX3 is involved in several RNA processes, it will be interesting to understand how DDX3 mediates its diverse functions and different processes, especially in the context of tumourigenesis.

## Supporting Information

S1 Fig DDX3 promotes the proliferation and migration of HCT116 cells. (A and B) CCK-8 assays of HCT116 colon cancer cells. The cells were transfected with pCMV-DDX3 and pCMV-2A (vector control) (A) or DDX3-specific siRNA (siDDX3) and siNC (negative control) with or without p65 stimulation (B). At 24 hours post transfection, the cells were seeded (3000 cells/well) in 96-well plates, which were incubated for 5 days in a humidified incubator with a 5% CO_2_ atmosphere at 37°C. Then, 10 μl of CCK-8 (5 mg/ml) (KaiJi, China) was added to each well, and the plates were incubated at 37°C for 1 hour. The number of cells was determined by measuring the absorbance at 450 nm using a microplate reader (BioTek, Winooski, Vermont). (C) In the cell migration assay, cells were transfected with siRNA (siDDX3 and siNC). At 24 hours post transfection, cells (1.5×10^5^) in FBS-free DMEM were added to the upper chamber, and DMEM containing 10% FBS was placed in the lower chamber. After 24 hours of incubation, the cells that did not migrate through the pores were removed using a wet cotton swab, and the cells on the lower surface were fixed with methanol and stained with 0.05% crystal violet. Finally, the cells were counted under a microscope, and the relative migration was calculated (lower panel). *n* = 3, mean ± SD. ** p < 0.01 and *** p < 0.001 (unpaired Student’s t-test).
(TIF)
