# Supplementary figures and images for: The DEAD-Box RNA Helicase DDX3 Interacts with NF-κB Subunit p65 and Suppresses p65-Mediated Transcription
Source: PLoS One. 2016 Oct 13;11(10):e0164471. doi: 10.1371/journal.pone.0164471 (PMC5063347; doi:10.1371/journal.pone.0164471)

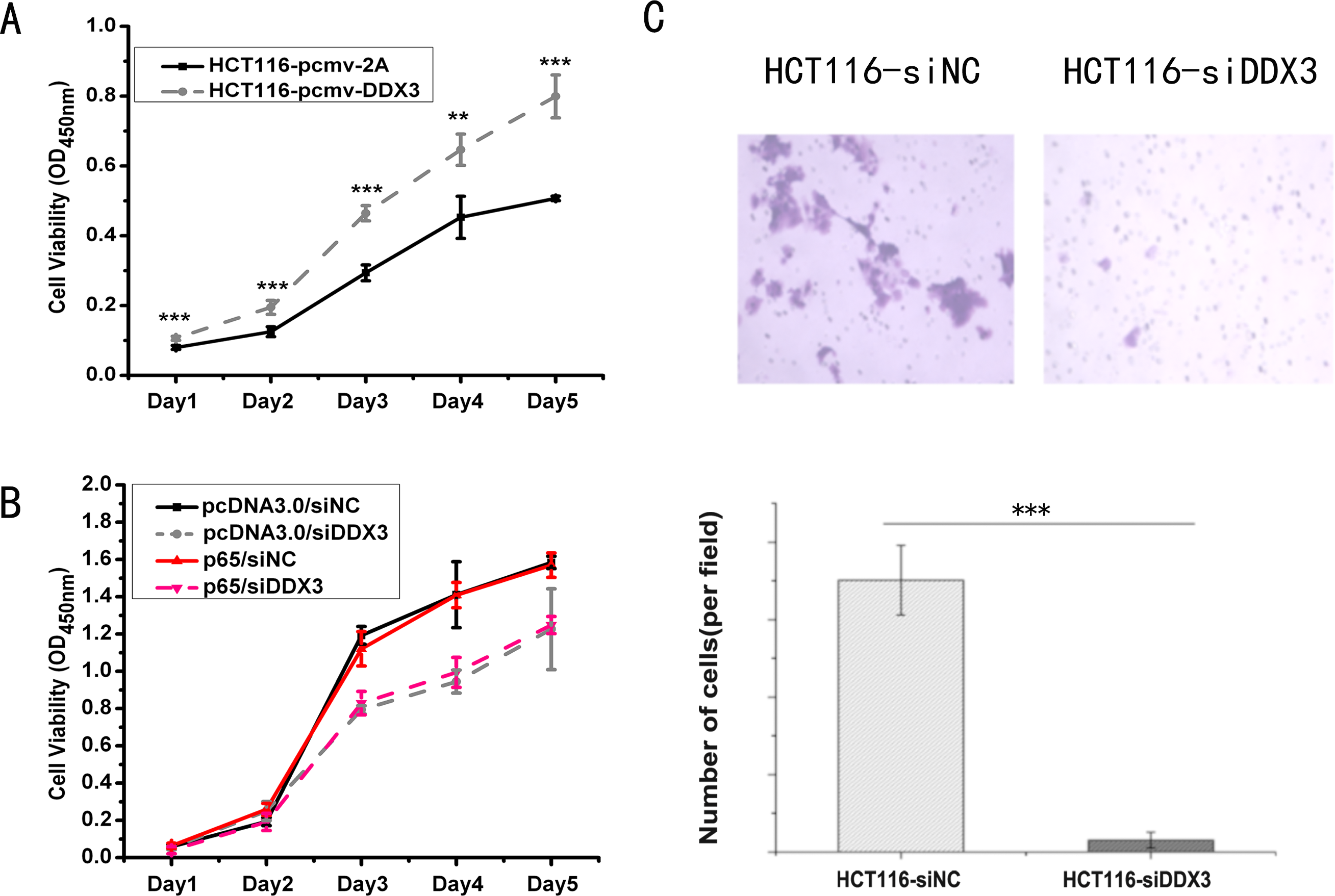

Supplement: S1 Fig — (A and B) CCK-8 assays of HCT116 colon cancer cells. The cells were transfected with pCMV-DDX3 and pCMV-2A (vector control) (A) or DDX3-specific siRNA (siDDX3) and siNC (negative control) with or without p65 stimulation (B). At 24 hours post transfection, the cells were seeded (3000 cells/well) in 96-well plates, which were incubated for 5 days in a humidified incubator with a 5% CO2 atmosphere at 37°C. Then, 10 μl of CCK-8 (5 mg/ml) (KaiJi, China) was added to each well, and the plates were incubated at 37°C for 1 hour. The number of cells was determined by measuring the absorbance at 450 nm using a microplate reader (BioTek, Winooski, Vermont). (C) In the cell migration assay, cells were transfected with siRNA (siDDX3 and siNC). At 24 hours post transfection, cells (1.5×10^5^) in FBS-free DMEM were added to the upper chamber, and DMEM containing 10% FBS was placed in the lower chamber. After 24 hours of incubation, the cells that did not migrate through the pores were removed using a wet cotton swab, and the cells on the lower surface were fixed with methanol and stained with 0.05% crystal violet. Finally, the cells were counted under a microscope, and the relative migration was calculated (lower panel). n = 3, mean ± SD. ** p < 0.01 and *** p < 0.001 (unpaired Student’s t-test). (TIF) [file pone.0164471.s001.tif]
